# Immunometabolic Checkpoints of Treg Dynamics: Adaptation to Microenvironmental Opportunities and Challenges

**DOI:** 10.3389/fimmu.2019.01889

**Published:** 2019-08-27

**Authors:** Ilenia Pacella, Silvia Piconese

**Affiliations:** ^1^Laboratory of Cellular and Molecular Immunology, Department of Internal Medicine and Medical Specialties, Sapienza Università di Roma, Rome, Italy; ^2^Laboratory Affiliated to Istituto Pasteur Italia – Fondazione Cenci Bolognetti, Rome, Italy

**Keywords:** Treg, glycolysis, cancer, proliferation, mitochondria, oxydation

## Abstract

In the last decades, immunologists have started to consider intracellular metabolism in relation with the dynamics and functions of immune cells, especially when it became clear that microenvironmental alterations were associated with immune dysfunctions. Regulatory T cells (Tregs) are equipped with a variety of immunological and metabolic sensors, and encompass circulating as well as tissue-resident cells, being therefore particularly susceptible to microenvironmental cues. Moreover, Tregs undergo metabolic reprogramming over the course of an immune response, allowing the use of alternate substrates and engaging different metabolic pathways for energetic demands. The study of metabolic mechanisms supporting Treg dynamics has led to puzzling results, due to several limitations, including the heterogeneity of population in the same tissues and between different tissues, the difficulty in considering all the interconnected metabolic pathways during a cellular process, and the differences between *in vitro* and *in vivo* conditions. Therefore, Treg reliance on different metabolic routes (oxidation rather than glycolysis) has been a matter of controversy in recent years. Metabolic reprogramming and altered bioenergetics are now identified as hallmarks in cancer, and are employed by cancer cells to determine the availability of metabolites and molecules, thus affecting the fate of tumor-infiltrating immune cells. In particular, the tumor microenvironment forces a metabolic restriction and a plethora of synergistic intrinsic and extrinsic stresses, leading to an impaired anti-tumor immunity and favoring Treg generation, expansion, and suppressive function. This leads to the understanding that Tregs and conventional T cells have different capability to adapt to metabolic hurdles. Considering the role of Tregs in dictating the outcome of tumor-specific responses, it would be important to understand the specific Treg metabolic profile that provides an advantage at the tumor site, to finally identify new targets for therapy. In this review, we will report and discuss the major recent findings about the metabolic pathways required for Treg development, expansion, migration and functions, in relation to tissue-derived signals. We will focus on the adipose tissue and the liver, where Tregs are exposed to a variety of metabolites, and on the tumor microenvironment as the context where Tregs develop the ability to adapt to perturbations in nutrient accessibility.

## Introduction

Regulatory T cells (Tregs) are now recognized as a specialized CD4 T cell subset essential for immune homeostasis, as well as for protection from autoimmunity and excessive inflammation. In several mouse models, transient Treg depletion early in life, or congenital Treg deficiency, leads to the spontaneous development of lethal multiorgan autoimmune disorders (1, 2). In humans, a wide array of Treg defects, ranging from frequency to function to proliferative potential, have been reported in several autoimmune diseases, and therapies aiming at recovering physiological Treg activities (such as adoptive Treg cell therapy) are under development for these conditions (3). Conversely, increased Treg proportions can be found in the tumor microenvironment in many tumor types in both mouse models and human patients, correlating with a poor prognosis [with a few exceptions like colorectal cancer (4)]. Therefore, Treg depletion or blockade is now considered as a necessary step to elicit effective anti-tumor immunity (5). Recent data have revealed that CTLA-4, the first “immune checkpoint” to enter the clinic as cancer immunotherapeutic agent, is more expressed by Tregs than effector T cells in peripheral lymphoid organs and in blood and even more at the tumor site, and that anti-CTLA-4 antibodies may work through antibody-dependent cell-mediated cytotoxicity and Treg depletion (6–9); this finding proves the key role of Tregs as a non-redundant and even dominant immune checkpoint in the tumor microenvironment. Therefore, a deeper understanding of the most important pathways and molecules involved in Treg expansion, survival and contraction is urgently needed in order to design better therapies aiming at Treg manipulation *in vivo*.

We have recently started to appreciate the complexity of Treg dynamics, from their development to their rapid adaptation to microenvironmental and systemic changes. More importantly, in the last decades, we have started to take into consideration intracellular metabolism of T cells in relation to their dynamics and functions during immune responses, a concept known as cellular “immunometabolism” (10). Tregs can be considered a very peculiar CD4 T cell subset, since they physiologically reside in virtually all tissues and organs, and constitutively express a wide array of immune as well as metabolic sensors. Therefore, Tregs are equipped to promptly respond to any immune and metabolic signal in an “innate-like” fashion, even though we have not completely elucidated the consequences of immunometabolic signals in Treg activities. Based on their high sensitivity to external cues and on their fundamental role in switching between tolerance and immunity, Tregs can be considered as one of the key links between nutrient sensing and immune response, a mechanism selected by evolution to optimize energetic resources (11).

## Tregs Switch Between Quiescence and Proliferation in Many Phases of Their Development and Functions

When CD25+ Tregs were discovered, they were originally described as anergic cells, based on their inability to proliferate *in vitro* in response to T cell receptor (TCR) stimulation and in the absence of exogenous IL-2 (12). What is more, anergy appeared as a prerequisite for suppressive function, since Tregs seemed to lose their suppression in condition of anergy reversal (12). Not only was anergy thought to be required for Treg function, but it seemed also involved in Treg differentiation. Indeed, since the very first experiments *in vitro*, the conversion of conventional T cells (Tconvs) into Tregs was favored in conditions of tolerogenic or sub-immunogenic stimulation that induced suboptimal levels of proliferation (13, 14). All these data contributed to consolidate the idea of anergy as a key component of the Treg identity. However, the observations that *de novo* induced Tregs can massively proliferate and that Tregs could preserve their suppressive function while proliferating have challenged this notion (14, 15). It is now recognized that, in many contexts, proliferation is not only involved but even required to ensure a full suppressive function by Tregs.

In the last decades, a large amount of data have clarified the requirement of active proliferation throughout the stages of Treg development and activation in mice, whilst the knowledge of these events remains still elusive in humans. A subset of Tregs, probably accounting for the majority of Tregs in lymphoid organs of naïve animals, develop in the thymus upon encountering self-antigens, and are called thymic Tregs (tTregs). A certain proportion of Tregs can develop in peripheral organs in response to non-self-molecules such as commensal and food antigens, are thus highly represented in the intestine, and are called peripheral Tregs (pTregs) (16). To date, no reliable markers are available to dissect the actual contribution of thymic vs. peripheral developmental routes to the Treg pool; however, several protocols have been developed to induce Treg differentiation *in vitro* (of so-called iTregs) from Tconvs, which recapitulate some features of Treg induction *in vivo*.

In the neonatal life, early after development, tTregs undergo a massive wave of proliferation that is probably their first proliferative burst. In mice, a distinct pool of Tregs largely expands in the perinatal life, persists longtime, and plays a vital role in suppressing autoimmunity (17) through the induction of T cell anergy (18). These perinatal Tregs presented very high proportions of Ki67-positive and EdU-incorporating cells, and DNA replication was one of the top pathways emerging from their transcriptomic profile. A similar expansion of Tregs has been detected in the human peripheral blood during early neonatal life, probably in response to the immediate exposure to commensals (19). Tregs display a phenotype that is compatible with recent activation in healthy human neonates, while during neonatal sepsis they may play a role in controlling the clinical manifestations of the disease (20).

Following development, Tregs recirculate throughout the blood and populate lymphoid as well as non-lymphoid organs; in the latter, tissue-resident Tregs acquire a tissue-specific molecular profile and specialized functions (21). This polarization from a central (cTreg) into an effector (eTreg) status occurs in response to antigen stimulation as well as local inflammatory stimuli, and follows the activation of specific molecular programs driven by transcription factors like NFkB, Blimp and IRF4 (22). Tissue-resident Tregs are thought to play not only the well-known immunosuppressive functions, but also to exert some non-immune activities such as maintenance of tissue homeostasis and promotion of tissue regeneration upon injury (23). Whether Tregs undergo further rounds of division within tissues, and whether this event is required to become specialized resident cells or to ensure their long-term persistence, is still unclear. Some tissues are characterized by relatively high frequencies and numbers of Tregs in physiological conditions, such as the bone marrow (24) and the visceral adipose tissue (VAT) of lean adult mice; at least in the latter case, it has been clearly demonstrated that active proliferation, coupled with enhanced survival, sustains the physiological Treg expansion in this tissue (25, 26). Several data confirm the idea that human circulating Tregs contain a very high proportion of cycling cells (27, 28) even though the role of proliferation in the physiological homeostasis of human Tregs remains to be clarified.

In pathological conditions, for instance following acute tissue injury, the Treg population may expand thanks to the concomitant recruitment of cTregs that are locally differentiated into eTregs, to the local development of pTregs, and to the proliferation of resident and recruited Tregs (29). Thanks to their proliferative response and effector polarization, Tregs can promote the resolution of the injury through the suppression of inflammatory processes and the release of tissue-repairing molecules. However, when the source of damage is not eradicated, such as in the case of viruses establishing chronic infections or in the case of cancer, the Treg pool may continue to expand in a chronic fashion, until it even subverts the protective anti-viral or anti-tumor immunity (30).

**Figure 1:**
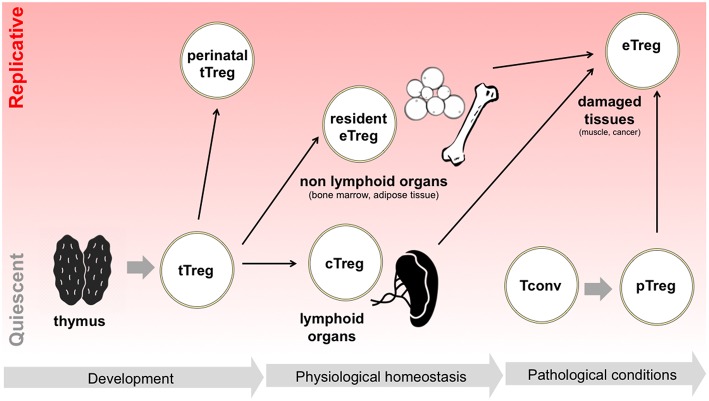
Tregs switch between quiescence and replication in different developmental and activation phases. Following tTreg development, a perinatal wave of Treg replication occurs, which is thought to suppress T cell responses in neonatal life. Later in life, in physiological conditions, Tregs colonize both lymphoid organs, becoming cTregs, and non-lymphoid organs, becoming resident Tregs and acquiring eTreg phenotype: in the latter case, extensive Treg proliferation has been reported, for instance in adipose tissue. Finally, in pathological conditions such as in the case of acute tissue damage, Tregs are locally expanded, thanks to the proliferation of resident eTregs, to the differentiation of cTregs, and to the conversion of pTregs.

All these pieces of evidence demonstrate that, during their life time, Tregs may continuously switch between quiescent survival and active replication and back; this switch may occur when Tregs are in a naïve status without compromising their “naiveness” as in the case of perinatal Treg expansion, or may occur during the physiological specialization of tissue-resident Tregs or the pathological polarization of effector Tregs in damaged tissues (Figure 1). Such highly dynamic behavior implies that Tregs may be particularly able to switch their metabolism depending on the immune signals that they receive and the nutrient availability in different contexts.

## The Metabolism of Conventional T Cells: How Do Tregs Fit With This Model?

Thanks to advances in the field in recent years, we have now recognized that T cells, probably the cells in the body capable of the largest clonal proliferation, strictly rely on metabolic switches to sustain their immune functions. Three main metabolic profiles can be generally assigned to the main stages of T cell activation: first, mature naïve T cells are thought to survive in a quiescent state thanks to a low level of oxidative phosphorylation and mitochondrial respiration; second, T cell stimulation through the TCR and costimulatory receptors primes a strong switch from an oxidative to a glycolytic-lipogenic metabolism, characterized by increased uptake of glucose (and other nutrients) from the extracellular environment, generation of ATP from substrate-level phosphorylation during glycolysis, conversion of pyruvate into lactate, and biosynthesis of macromolecules for cell growth and division; third, memory T cells are mostly oxidative cells, yet they are “metabolically primed” for a glycolytic switch, a combination that ensures both long-term survival and prompt response to an antigen recall (31, 32).

The glycolytic-lipogenic and the lipolytic-oxidative pathways are generally viewed as mutually exclusive, thanks to the reciprocal regulation operated at several checkpoints along the metabolic flux. As an example, in poor nutrient supply conditions and other stress conditions, the AMP-activated protein kinase (AMPK) is activated and negatively regulates the mammalian target of rapamycin (mTOR), thus inhibiting a variety of biosynthetic pathways such as fatty acid synthesis (mediated by acetyl-CoA carboxylase or ACC) and cholesterol synthesis. A reciprocal regulation also exists between fatty acid synthesis and oxidation, since malonyl-coA (the product of ACC reaction) is a major inhibitor of carnitine palmitoyltransferase 1a (or CPT1a, the key enzyme of fatty acid oxidation). Therefore, cells are thought to oscillate between an mTOR-driven anabolic metabolism and an AMPK-driven catabolic metabolism. However, the two programs have been found to coexist in selected conditions. Memory T cells, indeed, maintain the ability to uptake and convert glucose into fatty acids, and then perform cell-intrinsic lipolysis to fuel mitochondrial oxidation, in an apparently “futile” cycle whose implications have not been completely understood (33).

A correspondence between the immune status and the intracellular metabolism can be achieved thanks to many immune-metabolic links in signaling pathways in T cells: to mention just a few examples, TCR and CD28 stimulation directly activates mTOR, a major orchestrator of the glycolytic switch (34); CD28 primes mitochondrial fatty acid oxidation, thus driving proper memory development (35); the enzyme glyceraldehyde 3-phosphate dehydrogenase (GAPDH), when not engaged in glycolysis, represses post-transcriptionally IFN-γ expression (36); and the tumor necrosis factor receptor-associated factor 6 (TRAF6), an adaptor that can be activated by some receptors of the tumor necrosis factor receptor (TNFR) superfamily, fosters memory development through mitochondrial fatty acid oxidation (37).

How can Tregs be categorized according to this scheme? The answer is still unclear: indeed, some data describe Tregs as mostly oxidative cells, while other data demonstrate the need of glycolysis for some Treg activities. One of the main reasons explaining these conflicting results may be the type of Tregs (tTregs vs. pTregs) that is analyzed in different studies: on the one side, pTregs seem to differentiate when effector T cell activation, which relies on the glycolysis-lipogenesis pathway, is blocked. Conversely, tTregs seem to depend on this pathway for their proliferation and fitness, resembling effectors (38); however, both tTregs and pTregs can either remain quiescent or enter cell cycle depending on the surrounding signals, and this can profoundly affect their metabolism. Therefore, probably the most important reason dictating their metabolic requirements is the stage of development and/or activity at which Tregs are analyzed; indeed, opposite results can be obtained when analyzing iTregs/pTregs during their differentiation from Tconvs, or when analyzing already established Tregs (irrespective of their peripheral or thymic origin) during their active proliferation. In turn, Tregs can be studied directly *ex vivo* from many different tissues and sources, or after many different types of stimulation and culture *in vitro*; this experimental heterogeneity may profoundly affect the outcome of metabolic analyses. Finally, the CD25high or Forkhead Box P3 (Foxp3) + compartment is actually a heterogeneous mixture of different subsets, which include cycling as well as resting Tregs, and also cells with an unstable regulatory phenotype.

Here we will report and discuss the main findings regarding Treg metabolism, classified according to whether they refer to *de novo* Treg induction, to the proliferation of previously established Tregs, or to different Treg activities.

## Treg Induction, Proliferation and Function Rely on Distinct Metabolic Pathways

### Metabolic Pathways Involved in Treg Induction

The first evidence connecting Treg induction to an oxidative metabolism came from the study of Michalek et al., where iTregs were differentiated through the classical protocol based on transforming growth factor β (TGFβ) exposure, or through mTOR inhibition with rapamycin; in both settings, etomoxir (at 200 μM), known as an inhibitor of fatty acid oxidation, could suppress iTreg polarization (39). Compared to T helper subsets polarized *in vitro* with specific cytokine cocktails, TGFβ-induced iTregs expressed lower levels of the glucose transporter 1 (GLUT1), performed less glycolysis and more fatty acid oxidation, and were not affected by supplementation of exogenous fatty acids (39). Both iTregs and so-called “natural” Tregs contained higher levels of phosphorylated AMPK, and metformin administration increased Treg frequency *in vivo* (39).

Subsequently, several studies have contributed to generate the hypothesis that Treg differentiation relies on a switch from glycolytic-lipogenic to oxidative metabolism. A large amount of data derive from the analysis of iTreg polarization *in vitro*, induced through standard protocols of anti-CD3/anti-CD28 stimulation in the presence of TGFβ. In this setting, iTregs display lower glycolytic rates and higher oxygen consumption compared to T helper (Th) 1 and Th17, and their metabolic and transcriptional profile is suggestive of higher fatty acid oxidation (40–44). In these cultures (actually containing Foxp3+ and Foxp3- cells in some studies), the differentiation of iTregs was reduced in the presence of the electron transport inhibitor rotenone (irrespective of their proliferation) (43); conversely, the glycolysis inhibitor 2-deoxy-D-glucose (2-DG) or the mTOR inhibitor rapamycin (45), or the AMPK agonist 5-aminoimidazole-4-carboxamide ribonucleotide (AICAR) (46, 47), enhanced iTreg differentiation. On the side of lipogenesis, the genetic or pharmacological blockade of fatty acid synthesis enhanced iTreg polarization at the expense of Th17 cells (42).

Key metabolic “checkpoints” have been identified that affect iTreg differentiation by tipping the balance between glycolytic and oxidative metabolism. The first step of this axis is represented by glucose uptake, operated by several glucose transporters and in particular by GLUT1. When its expression was ablated in the T cell lineage, effector T cells displayed defective growth, proliferation and survival; however, Treg numbers were not affected, either in naïve mice or under inflammatory conditions (48). Downstream glucose capture, mTOR is a major sensor of environmental cues including immune signals and nutrient availability, and is also a major orchestrator of the glycolytic-lipogenic switch required for cell growth and proliferation; not only mTOR inhibition enhances iTreg differentiation (49, 50), but also mice carrying T cell-restricted mTOR deficiency show impaired T helper cell expansion, with enhanced Treg induction, *in vitro* and *in vivo*, in a model of viral infection (51). Downstream mTOR, the transcription factor HIF1α is activated and initiates a glycolytic program in T cells that is required for Th17 polarization at the expense of iTreg induction (45, 52). Pyruvate dehydrogenase (PDH), whose activity is negatively regulated by the pyruvate dehydrogenase kinase 1 (PDK1), dictates the fate of pyruvate between conversion into lactate or into acetyl-CoA; this axis was identified as a key regulatory node for Th17 and iTreg alternative polarization *in vitro* (43). Finally, the acetyl-coA carboxylase 1 (ACC1), a key enzyme for fatty acid synthesis, was shown to switch the polarization fate between iTregs and Th17 (42).

These findings strengthened the notion that iTreg/pTreg differentiation was favored in conditions of oxidative metabolism and was antagonized when the glycolytic-lipogenic pathway was fueled. However, some considerations should be made when interpreting these results, especially the studies performed in mouse models. First, most data come from mice carrying genetic ablation of key genes in the entire T cell lineage, thus effector T cell development, homeostasis and activation may be suboptimal or defective. In these settings, some of the Treg alterations, especially those observed *in vivo*, may be a consequence of impaired effector T cell homeostasis or activation, rather than regarding selectively iTreg/pTreg induction. Second, most data have been obtained in contexts of antigen immunization, autoimmune or inflammatory diseases: for instance, Foxp3+ cells (probably a mixture of tTregs and pTregs) did not upregulate proteins related to glycolysis, contrary to Th17 cells, in lymphoid organs of mice with experimental autoimmune encephalomyelitis (EAE) (43). Other examples are that administration of 2-DG and metformin increased the frequency of Foxp3+ cells in splenocytes of mice immunized with a model antigen (53), and that mice lacking HIF1α or ACC1 in the CD4 T cell lineage showed higher Treg frequencies and were more resistant to EAE (42, 52). These inflammatory conditions generally do not favor massive induction of expansion of Tregs, and thus do not allow studying Treg metabolism in a dynamic context. Third, discriminating the metabolic requirements of newly induced pTregs, compared to preexisting tTregs undergoing activation during disease, may be quite difficult *in vivo*.

**Figure 2:**
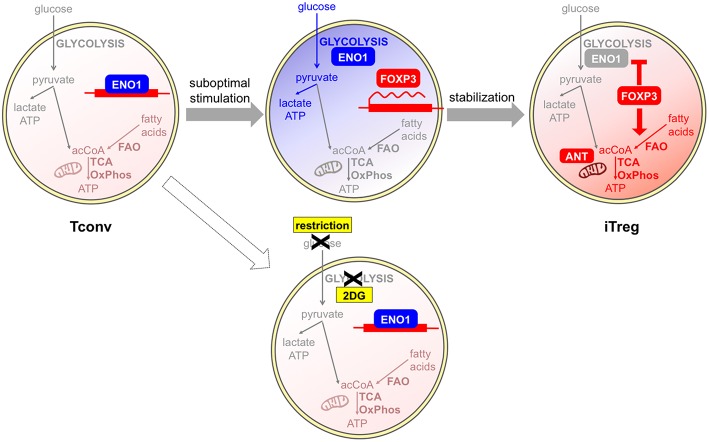
Glycolysis and oxidation may be involved in different phases of iTreg development. Tconvs show a basal metabolism that is based on fatty acid oxidation (FAO) and the usage of acetyl CoA (acCoA) to fuel the TCA cycle and oxidative phosphorylation (OxPhos). Human Tregs can be differentiated *in vitro* from Tconvs following suboptimal antigen stimulation. This challenge induces a shift from the basal oxidative (light red) to the glycolytic (blue) metabolism: as a consequence, the glycolytic enzyme enolase-1 (ENO1) is engaged in the glycolytic cascade and thus displaced from the FOXP3 locus, where the FOXP3 gene can be transcribed. If glycolysis is blocked by a pharmacological inhibitor, or is impeded in conditions of glucose restriction, enolase-1 continues to repress FOXP3 transcription and iTreg development cannot occur (55). At later stages, FOXP3 itself can induce a stabilization of the iTreg program and a rescue of a quiescent state, through the enforcement of mitochondrial metabolism (red) that is mediated by an upregulation of mitochondrial proteins (44). A proper mitochondrial function is under the control of the ANT transporter and is required for iTreg induction (47).

Regarding the *in vitro* studies, most conclusions have been drawn from experiments based on the classical protocol of TGFβ culture. In this setting, iTreg induction appears as a “backup plan” when other differentiation programs are not allowed. Since T helper polarization usually requires T cell growth supported by a glycolytic-lipogenic switch, T cells may sense metabolic restriction as a signal of functional anergy, which consequently leads to Foxp3 induction and initiates a regulatory program. Supporting this idea, the increase of iTreg polarization induced by some metabolic inhibitors like the ACC1 inhibitor soraphen A, the mitochondrial blocker rotenone (41–43), or appearing in mTOR-deficient cells (51), was coupled to a suppression of T cell proliferation; conversely, higher T cell proliferation was induced by the AMPK agonist AICAR along with lower iTreg induction (46). Therefore, these results suggest the existence of a link between metabolic and immune anergy, of which Foxp3 induction may be an early consequence (54). At later stages, Foxp3 may stabilize this profile; indeed, ectopic Foxp3 expression is necessary and sufficient to induce a switch from glycolysis to oxidation (40, 43, 44) (Figure 2).

Recent data have clearly demonstrated that TGFβ has the intrinsic property of suppressing glycolytic metabolism in Tconvs undergoing transition into iTregs, as well as in tTregs, which would be otherwise highly glycolytic cells (56). Therefore, TGFβ-based protocols for iTreg polarization may provide limited information on the physiological mechanisms of iTreg differentiation. Indeed, strikingly different results have been obtained when human iTregs have been differentiated *in vitro* in a TGFβ-independent fashion, under conditions of suboptimal (weak and short) CD3/CD28 stimulation (55). In this setting, a population of CD25high cells developed, enriched in Ki67+ and FOXP3+ cells, in which the induction of high levels of FOXP3 was suppressed by 2-DG and enhanced by etomoxir. Two splicing isoforms of the human FOXP3 protein exist differing for the presence of the exon 2 and conferring different suppressive properties and lineage stability: the glycolytic enzyme enolase-1 was found recruited to the FOXP3 locus to repress FOXP3 expression, especially when 2-DG treatment inhibited glycolysis thus disengaging glycolytic enzymes. Impaired glycolysis and diminished expression of the FOXP3 isoform containing exon 2 were observed in Tconvs obtained from multiple sclerosis patients in correlation with their lower rates of conversion into iTregs (55). This study challenged the idea that iTreg differentiation was antagonized in conditions of high glycolytic rates; rather, it demonstrated that a proper metabolic activation of Tconvs, which included competence for glycolysis, was a prerequisite for proper iTreg development and that the metabolic requirements for iTreg differentiation were not antagonistic but rather parallel to the metabolic requirements for effector T cell activation. Based on this view, it could be hypothesized that regulation is allowed to develop along with immunity in conditions of optimal nutrient availability and competence for nutrient usage (Figure 2). Also in mouse models, weak TCR signal strength has been proven to preferentially promote Treg induction and/or expansion (13, 14, 57); however, one study has shown that low-dose antigen stimulation was not associated with strong mTOR activation (57). Therefore, differences in mTOR involvement and metabolic requirements may exist between human and mouse Treg induction under weak antigen stimulation.

Some compounds that have been used in many immunometabolism studies, such as 2-DG and etomoxir, have displayed some “off-target” effects that were not recognized before. In macrophages, 2-DG has been shown to block not only glycolysis but also oxidative phosphorylation and ATP production (58). Etomoxir, when used at low doses, blocks the oxidation of long-chain fatty acids through the inhibition of CPT1a, the rate-limiting enzyme for this process. However, when used at high doses, it can block mitochondrial respiration directly, irrespective of the nutrient (glucose, glutamine or fatty acids) fueling oxidative phosphorylation in T cells (47). Also in macrophages, high concentrations of etomoxir can display CPT1a-independent effects that are mediated by the depletion of intracellular free coenzyme A (59). Therefore, results obtained with the use of these pharmacological inhibitors *in vitro* should be interpreted with caution, while approaches based on the genetic ablation of key metabolic enzymes may probably shed light on the exact metabolic requirement for Treg differentiation (60). Accordingly, when CPT1a was genetically abolished selectively in the T cell lineage, TGFβ-driven iTreg polarization *in vitro* was not affected, and Treg frequency was found to be normal *in vivo* in these mice in physiological conditions. Instead, etomoxir at high doses suppressed iTreg polarization through the inhibition of adenine nuclear translocator (ANT), a transporter that affects ATP concentration in the mitochondrial matrix, the mitochondrial membrane potential, and the activity of the electron transport chain (47). These data questioned the assumption that fatty acid oxidation was a driving force for iTreg/pTreg differentiation. Together with data from De Rosa et al. (55), these results support the idea that Treg development from converting T cells may require the optimal activation of multiple routes, which may not involve long chain fatty acid oxidation but does include mitochondrial respiration.

### Metabolic Pathways Involved in Treg Homeostasis and Proliferation

**Figure 3:**
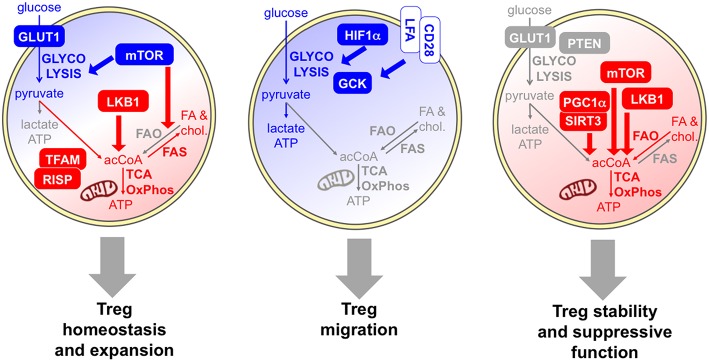
Specific metabolic pathways and key molecules are involved in distinct Treg activities. Several data indicate that Tregs rely on a combination of glycolytic (blue) and mitochondrial (red) metabolism for their homeostasis and expansion. Proliferative Tregs express GLUT1 and constitutive mTOR (61, 62), and Tregs require an mTOR-dependent glycolysis-lipogenesis for their expansion (63). Indeed, fatty acid synthesis (FAS) and cholesterol synthesis occur. LKB1 is required for Treg development and survival through the support of mitochondrial function (64, 65). Evidence that mitochondrial activities are required for Treg fitness comes from experimental models of deficiency of mitochondrial complex III (RISP) (66) or mitochondrial transcription factor A (TFAM) (67). However, FAO may not be required for Treg development (47). The key role of glycolysis (blue) in Treg migration is demonstrated by the evidence that HIF1α-deficient Tregs show lower glycolysis and display impaired migratory ability *in vitro* and *in vivo* (68), and by the observation that pro-migratory stimuli, like LFA1 or CD28, enhanced glucose uptake and glycolytic rate while promoting migration, through the induction of the glycolytic enzyme GCK (69). Regarding Treg suppressive function and stability, some data indicate a glycolytic boost may compromise Treg suppressive function and stability *in vivo*: this event occurs in mice lacking PTEN selectively in the Treg lineage (70) and in transgenic mice overexpressing GLUT1 (62). Other studies support the idea that mitochondrial activity (red) is required for Treg suppressive function: key molecules involved in this regulation are SIRT3/PGC1α (41) the kinase LKB1 (64, 71), and mTOR, which promotes the transition from cTregs into eTregs via mitochondrial activation (67).

As summarized above, several findings indicate that Tregs actively proliferate during their lifetime and that the Treg population may comprise a high proportion of cycling cells, from many districts, in physiological and pathological conditions. Their hyporesponsiveness *in vitro*, which has been interpreted as a functional and metabolic “anergy,” may rather derive from a functional and metabolic activated status, making them refractory to strong stimulation in culture (11). Thus, it is reasonable to suppose that Tregs rely on a glycolytic-lipogenic metabolism for their fitness and their proliferative bursts (Figure 3).

Several data regarding freshly isolated human and mouse Tregs (likely comprising a mixture of tTregs and pTregs) confirm this hypothesis. Procaccini et al. have shown that, contrary to conventional T cells, Tregs show constitutively active mTOR directly *ex vivo*: a transient mTOR inhibition, achieved through rapamycin administration or through nutrient starvation and interruption of the leptin/leptin receptor signal, turns these cells from anergic into highly proliferative cells both *in vitro* and *in vivo*. Thus, it has been proposed that an “oscillatory” mode of mTOR activity is responsible for the exit of Tregs from a hyporesponsive into a proliferative status (61). These findings have been confirmed also by Gerriets at al. who showed that Ki67 high Tregs from the spleen of naïve mice express higher levels of GLUT1 and mTOR activity (62). A proteomic and biochemical profile of freshly extracted human Tregs has revealed that these cells are highly glycolytic *ex vivo* and utilize both glycolysis and fatty acid oxidation for their proliferation *in vitro* (as assessed with the use of 2-DG or etomoxir). In contrast, effector T cells are mostly oxidative *ex vivo* but rely on glycolysis when cultured *in vitro* (72). In line with these findings, others have found that freshly extracted human Tregs express high levels of genes related to glycolysis and lipid metabolism, and capture glucose at high efficiency (73, 74).

The pivotal role played by mTOR in Treg expansion has been demonstrated in several models. Mice carrying the Treg-intrinsic ablation of mTORC1 or mTOR spontaneously developed a severe scurfy-like autoimmune and inflammatory systemic disease (63, 67). Even if Treg numbers appear normal in mice with Treg-restricted mTORC1 deficiency, these cells displayed a severely impaired competitive fitness *in vivo*, which was accompanied by a decreased glycolytic rate, and which explained their defective suppression at the systemic level (63). Notably, the cholesterol synthesis was the most affected pathway by mTORC1 deficiency, and mTORC1-deficient Tregs failed to incorporate efficiently glucose-derived carbons into lipids; these findings demonstrate that Tregs utilize an mTOR-dependent glycolysis-lipogenesis for their expansion (63).

Much effort has been made to elucidate the role of fatty acid oxidation and mitochondrial metabolism in Treg expansion and homeostasis. Liver kinase B 1 (LKB1) is a bioenergetic sensor that phosphorylates AMPK and thus triggers oxidative catabolism, thus allowing cell survival under stress conditions. Mice lacking LKB1 specifically in Tregs display spontaneous type-2 inflammatory disease caused by defective Treg development and survival (64, 65, 71). Notably, LKB1-deficient Tregs display transcriptomic and metabolomic profiles compatible with impairment in tricarboxylic acid cycle, mitochondrial function and fatty acid oxidation (64, 65). The activity of LKB1 was not mediated by AMPK, since the Treg-restricted deficiency of AMPKα1 and AMPKα2 did not affect Treg numbers and did not induce any spontaneous inflammatory disorder (64, 65). Fatty acid oxidation was also not required for the physiological Treg development; according to data obtained in mice bearing a genetic CPT1a deficiency specifically in Tregs, CPT1a was dispensable for Treg development, Foxp3 expression, and suppressive function (47). Therefore, the AMPK-driven fatty acid oxidation does not seem to be involved in Treg homeostasis in physiological conditions.

When the mitochondrial complex III was genetically ablated in Tregs, a scurfy-like disease was observed, characterized by apparently normal Treg numbers but defective Treg competitive fitness and Treg suppression at the systemic level (66). Similar findings have been obtained in mice bearing a Treg-specific deletion of mitochondrial transcription factor A (TFAM), which is essential for electron transport chain activity: here a specific defect in eTreg development was identified (67). In complex III-deficient mice, the Treg defect was accompanied by diminished oxygen consumption, concomitantly increased glycolysis, and lower NAD+/NADH ratio (an index of the electron transport chain activity). However, the metabolic alterations observed in these mice might affect Tregs indirectly, through a novel metabolic-epigenetic circuitry; loss of mitochondrial complex III results in higher content of two metabolites, 2-hydroxyglutarate and succinate, known to inhibit DNA demethylases, and indeed complex III-deficient Tregs show DNA hypermethylation and altered gene expression (66).

### Metabolic Pathways Involved in Treg Activities: Migration, Suppression, and Stability

In both physiological and pathological conditions, circulating or lymphoid Tregs can undergo further functional and metabolic reprogramming while accomplishing their functions: indeed, Tregs can be attracted into specific tissues where they can experience further rounds of proliferation and/or functional specialization, can exert specific activities such as immune regulation and tissue repair, and can corroborate or instead destabilize their regulatory program, depending on the microenvironmental signals (29, 75, 76). Whether Tregs need to activate specific metabolic pathways to accomplish each of these tasks has not been completely elucidated. However, many studies (mentioned below) suggest that some “division of labor” may exist between different metabolic pathways in Tregs: on the one side, glucose usage and glycolysis are required for migration and rather antagonize Treg stability and suppressive function; on the other side, oxidative phosphorylation may be needed for Treg immune suppressive function (Figure 3).

The role of glycolysis in Treg migration has been demonstrated in several settings. Compared to wild-type Tregs, HIF1α-deficient Tregs show lower glycolysis and higher oxidative phosphorylation of both fatty acids and glucose, when cultured in normoxic or hypoxic conditions; functionally, HIF1α-KO Tregs suppress CD8 T cell proliferation *in vitro* with higher potency under hypoxia but display impaired migratory ability *in vitro* and *in vivo*. In mice with a Treg-restricted HIF1α deficiency, the reduced Treg recruitment to the site of a growing glioma leads to significantly delayed tumor growth and longer survival (68). This study suggests that HIF1α, and thus oxygen tension in the microenvironment, may dictate the balance between migration, relaying on glycolysis, and Treg suppressive function, at least *in vitro*. However, it does not seem to affect the physiological Treg development in the thymus, Treg peripheral fitness, or Treg proliferation *in vitro* or *in vivo*, probably because these processes occur in normoxic microenvironments. Kishore et al. have demonstrated the role of glycolysis in Treg migration and dissected the underlying molecular mechanisms: pro-migratory stimuli, like engagement of Lymphocyte function-associated antigen 1 (LFA-1) or of the costimulatory molecule CD28, enhanced glucose uptake and glycolytic rate while promoting migration. These events were mediated by mTORC2 and culminated in the induction of glucokinase (GCK), a hexokinase isoenzyme that interacts with actin and acts as a glycolytic ATP supplier for cytoskeletal rearrangements and cell migration (69). Notably, GCK-silenced Tregs did not show any defect in proliferation or suppression *in vitro*, and their compromised suppressive function *in vivo* could be ascribed to defective recruitment to the inflamed site. These results support the hypothesis of a dichotomy between glycolysis and oxidative phosphorylation being required for distinct activities of Tregs, migration and suppressive function, respectively.

Some pieces of information support the idea that a glycolytic boost may selectively compromise Treg suppressive function and stability *in vivo*, apparently without affecting their development. Mice lacking Phosphatase and tensin homolog (PTEN) selectively in the Treg lineage spontaneously develop systemic lymphoproliferation and lupus-like disease with age, despite increased Treg frequencies. Indeed, PTEN-deficient Tregs lost their suppressive function *in vivo* in the EAE model and displayed signs of functional instability, i.e., the release of pro-inflammatory cytokines in the inflamed site (70). Notably, this “fragile” phenotype correlated with higher glycolytic rates (but normal oxidative profile) of PTEN-KO Tregs compared to control Tregs, at short time (70) but not at later time points (56) after *in vitro* activation. Similar conclusions have been drawn from the analysis of Tregs in transgenic mice overexpressing GLUT1: also in this model, which develops again spontaneous autoimmunity, Tregs were more glycolytic, were expanded in numbers, but were less suppressive and more fragile *in vivo* (62). It may be speculated that glycolysis may promote the proliferation of Tregs that, instead of performing classical immune suppressive functions, are skewed toward a tissue-repair program, which may become relevant in specific conditions and microenvironments *in vivo*. It is important to consider that Treg uptake of glucose may induce T cell suppression by itself, irrespective of the Treg-intrinsic metabolic pathways. Indeed, competition for glucose is a key element for T cell activation especially under glucose restriction, and the proficiency of Tregs to internalize glucose leads to an induced senescence in surrounding T cells (74).

Conversely, other studies support the idea that mitochondrial activity may be required for Treg suppressive function. Tregs have greater mitochondrial mass and reactive oxygen species (ROS) production than conventional T cells, and Tregs lacking sirtuin 3 (SIRT3) or peroxisome proliferator-activated receptor gamma coactivator 1-alpha (PGC1α), both essential for mitochondrial activities, display weakened suppressive functions *in vitro* and *in vivo* (41). The deletion of histone deacetylase 9 (HDAC9) increases in Tregs the expression of genes related to oxidative phosphorylation (including SIRT3 or PGC1α), enhancing mitochondrial respiration; these cells also display increased suppressive function *in vitro* (41). Further studies have identified the complex I of the electron transport chain as an important element for Treg suppressive function (40). The kinase LKB1, which induces a transcriptional program oriented to mitochondrial metabolism (64, 65), is not only required for proper Treg development and survival, but also implicated in maintaining Treg stability and suppressive function (64, 71), further supporting the idea that proficiency for oxidative phosphorylation may be a prerequisite for Treg suppression. Recent data have revealed that mTOR not only drives Treg expansion through a glycolytic-lipogenic program (63), but may also support Treg suppressive function promoting the transition from cTregs into eTregs, via mitochondrial activation (67). *In vitro*, Treg suppressive function was reduced if Tregs were preactivated under acute pharmacological mTOR inhibition; *in vivo*, the acute deletion of mTOR in Tregs led to spontaneous loss of immune tolerance (67). Notably, mTOR activation was required for the acquisition of an eTreg phenotype through the post-transcriptional regulation of Interferon regulatory factor 4 (IRF4) expression. Following *in vitro* activation, Tregs significantly upregulated genes associated with mitochondrial metabolic pathways such as the tricarboxylic acid (TCA) cycle and the electron transport chain, in an mTOR-dependent fashion. It should be noted that, in many experimental systems including the mTOR-deficient mouse models, it might be quite difficult to discriminate between metabolic pathways involved in Treg expansion/fitness and in Treg effector function, since these two events are often coupled. Therefore, functional links between metabolic deficiencies and Treg alterations should be interpreted with caution.

## Main Immune Signals and Factors Controlling Treg Metabolism

Foxp3 is recognized as the major transcription factor underlying Treg identity; however, Foxp3 expression alone may not be sufficient for a stable and complete Treg differentiation and function, which also require continuous TCR stimulation and epigenetic reprogramming (77). Some metabolic pathways have been selectively linked to Foxp3 activity. Indeed, it has been shown that ectopic Foxp3 expression is necessary and sufficient to increase the expression of genes involved in mitochondrial respiration and to enhance fatty acid oxidation; in turn, the ability to catabolize fatty acids protects Foxp3-expressing cells from lipotoxicity, an event that may promote the selection of this population in mixed cultures and in stressed microenvironments (44). Foxp3 expression not only promotes oxidative metabolism but also suppresses glycolysis through the inhibition of MYC, a key factor of T cell metabolism (78), and also favors the oxidation of L-lactate to pyruvate through the modulation of lactate dehydrogenase (LDH) (40, 62). Therefore, Foxp3 expression may prime a default metabolic program that is shifted from glycolysis to oxidation.

**Figure 4:**
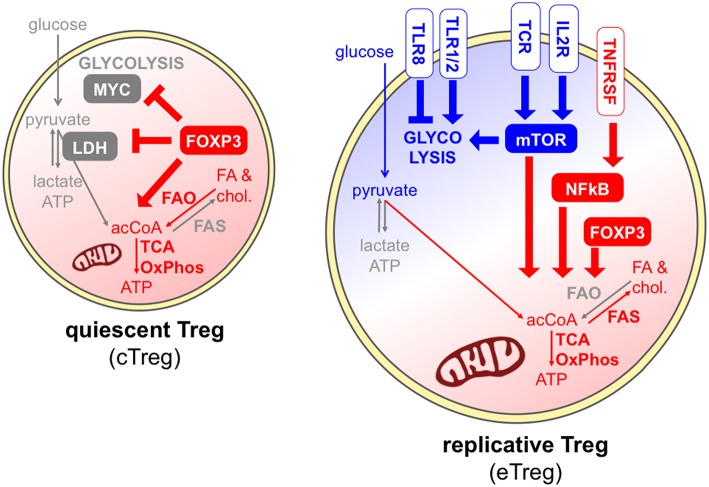
Different signals can promote or antagonize Treg exit from quiescence. In quiescent Tregs, Foxp3 maintains Treg survival through the promotion of fatty acid oxidation and mitochondrial metabolism (red) (44), and the suppression of glycolysis through the inhibition of MYC and the modulation of LDH (40, 62). Effector Tregs, undergoing cell division, can receive multiple signals through the TCR, costimulatory and cytokine receptors, and mostly display a combination of glycolytic (blue) and mitochondrial (red) metabolism. IL-2 receptor and the TCR can directly promote mTORC1 activity (63), and IL-2 signal supports cholesterol biosynthesis and mitochondrial activity through mTOR (79). A key role for mTOR-driven activation of mitochondrial metabolism has been identified in eTreg differentiation (67). However, FAO may not be required for Treg activation (47). Receptors of the TNFR superfamily activate NFkB thus inducing the eTreg phenotype (22), and this may occur also through the priming of mitochondrial functions, similarly to CD28 (35). Finally, different TLRs can have opposite functions on Treg glycolytic activities and proliferation (73, 80).

Contrary to conventional T cells, Tregs constitutively express a variety of costimulatory, inhibitory, and cytokine receptors, playing diverse roles in Treg maintenance and functions and also impacting on Treg metabolism (Figure 4). The IL-2 receptor conveys indispensable signals for Treg thymic expansion and maturation and for Treg peripheral homeostasis (79). Together with the TCR, IL-2 represents one of the predominant signals that promote mTORC1 activity (63); accordingly, peripheral Tregs lacking the high affinity IL-2 receptor displayed a transcriptional program compatible with reduced cholesterol biosynthesis and also disrupted mitochondrial activity (79). Several receptors belonging to the TNFR superfamily, like TNFR2 and OX40, are also constitutively expressed by Tregs, are massively upregulated following Treg activation, and convey key signals for the NFkB-mediated acquisition of an eTreg phenotype (22). A key role for mTOR-driven activation of mitochondrial metabolism has been identified in eTreg differentiation (67). Whether the TNFR-NFkB axis promotes the conversion of cTregs into eTregs through the priming of mitochondrial functions, similarly to the pathway described for another costimulatory molecule, CD28 (35), remains to be understood.

Tregs express a series of Toll-like receptors (TLR), and their stimulation may subvert or promote Treg suppressive and stability depending on the TLR type (80). Gerriets et al. showed that TLR1/TLR2 ligation on murine activated iTregs boosted their proliferation and their glycolysis, but also compromised their suppressive ability (62). This event may explain the destabilization of Tregs occurring at inflamed sites where TLR ligands are abundant, and further corroborates the idea that certain strong inflammatory signals may uncouple glycolysis-related proliferation and oxidation-related immune suppression. Opposite results have been obtained when TLR8 was stimulated in human Tregs: this treatment subverted their suppressive functions, however this event was accompanied by a loss, and not a gain, of GLUT-mediated glucose uptake and mTOR-dependent glycolytic activities (73). Therefore, different TLRs may operate completely divergent functions in Treg expansion and suppression, which may involve opposite metabolic rewiring.

## Extracellular Fatty Acids: Nutrients or Signaling Molecules?

Beside internalizing glucose from the outer environment and oxidizing intracellular lipids, T cells can capture and catabolize other molecules as a source of energy and biosynthetic precursors, such as the amino acid glutamine that is processed through glutaminolysis and fuels the TCA cycle, and plays a role in the reciprocal regulation of Th17 and iTreg differentiation (81).

Free long-chain fatty acids represent another potential extracellular nutrient for T cells, following their internalization via specific translocators, such as CD36. Several studies have reported that Tregs can capture fatty acids in culture (39, 42, 44) and also *in vivo*, especially in the tumor microenvironment in a mouse model of glioma (82). Notably, palmitate internalization and glucose uptake occurred in two distinct subpopulations of Tregs, with only minor overlap (82). In the VAT, Tregs acquire a tissue-specific program driven by the transcription factor peroxisome proliferator-activated receptor gamma (PPARγ) (83). This factor controls the expression of genes related to fatty acid translocation, biosynthesis, and oxidation; treatment with a PPARγ-agonist induced CD36 upregulation and fatty acid uptake in adipose tissue Tregs, and this event was associated with Treg expansion (83).

The relevance of long-chain fatty acid uptake in Treg differentiation and functions is still unclear. *In vitro*, exogenous BSA-conjugated palmitate was incorporated at high levels into endogenous fatty acids in iTregs, and the inhibition of cellular synthesis enhanced the extracellular fatty acid uptake (42). Supplemented palmitate enhanced oxidation rates in cells ectopically expressing Foxp3 (44) and iTregs *in vitro*, leading to a skewed iTreg development at the expense of Th17 cells (39, 84). Of note, the Foxp3-driven oxidative machinery may protect Tregs from the risk of lipotoxicity induced by high rates of fatty acid internalization (44). Based on these findings, exogenous fatty acids seem to promote iTreg polarization even though BSA-conjugated fatty acids may be internalized irrespective of physiological translocation that is mostly mediated by CD36 and other transporters.

Other data indicate that fatty acid uptake may also favor Treg suppressive function; indeed, the inhibitor of fatty acid translocation, sulfo-N-succinimidyl oleate (SSO), altered the expression of key suppressive molecules of Tregs and their inhibitory activity *in vitro* (68). As a general interpretation, high concentration of free fatty acids in a certain microenvironment may tip the balance toward immune regulation, even though the exact role of this metabolic-immune crosstalk in modulating immune responses deserves further studies.

Extracellular short-chain fatty acids (acetate, butyrate and propionate) exert a well-established role in Treg differentiation and expansion in the intestine; several studies [reviewed in Zeng and Chi (85)] have demonstrated that bacterial species colonizing the gut can break down dietary fibers, thus leading to the production of short-chain fatty acids. These metabolites bind specific G protein-coupled receptors on the cell surface and can promote the conversion of conventional T cells into pTregs or induce the proliferation of colonic tTregs. Therefore, short-chain fatty acids act as a bridge between microbial/dietary metabolism and immune regulation by working as signaling molecules, and less likely by directly affecting intracellular metabolic routes.

## Treg Expansion in Tissues Devoted to Metabolic Functions: the Adipose Tissue and the Liver

Since nutrient availability and signaling molecules can impact on Treg cell-intrinsic metabolism, which in turn can dictate Treg expansion and function, it could be predicted that the metabolic context of specific tissue microenvironments may affect immune regulation. This event may become particularly true in those tissues that are devoted to the control of systemic metabolism, such as the VAT and the liver. Presumably, in these tissues, the concentration of particular nutrients in the extracellular environment may vary depending on systemic and local metabolic processes.

Several groups have observed a prominent Treg accumulation in the VAT of healthy lean mice, have characterized the main mechanisms driving their expansion and suppressive function, and have reported a causal link between VAT-Treg deficiencies and metabolic inflammation (26, 83, 86–88). Among the signals driving VAT-Treg accumulation in mice, the TCR, Foxp3, and cytokines like IL-33 seem to play key roles (25, 26, 87). While these studies have demonstrated that Tregs can shape systemic metabolism through the suppression of metabolic inflammation in the VAT, it is not clear whether the local metabolism can affect Treg behavior. As already mentioned above, in the healthy VAT, PPARγ promotes the accumulation of intracellular lipids in Tregs; in obese mice fed with a high-fat diet, the reduction of Treg frequency corresponds to lower lipid content in Tregs (83). This result suggests that intracellular lipid accumulation may occur in alternate conditions in adipocytes (under dietary fat overload) and in Tregs (in healthy conditions), and also indicates that Tregs may not accumulate lipids as a simple consequence of extracellular or systemic lipid abundance. Less is known about Treg accumulation in the VAT of lean or obese humans and of patients with metabolic inflammation or cancer. According to recent data, Tregs express PPARγ also in the omental adipose tissue of humans, and Treg frequency is moderately increased in that tissue compared to the peripheral blood; however, no significant variation was observed in obese subjects or in type-2 diabetes patients compared to healthy controls (89). We observed even an expansion of Tregs in the VAT of obese compared to control subjects, and a mild positive correlation between VAT-Treg frequency and body mass index (90). Therefore, the mechanisms that regulate Treg accumulation in VAT may be completely distinct in mice and humans. Interestingly, we could observe a positive correlation between VAT-Treg percentage and the amount of the polyunsaturated ω6 arachidonic acid in the adipose tissue (90). Whether Tregs and generally immune cells can somehow shape the lipid composition of the tissue, and whether, conversely, different types of fatty acids can have an impact on Tregs and immune populations, remain open questions. It is worth noting that, in mice, VAT-Tregs utilize a catabolite of prostaglandin-E2 (which is synthetized from arachidonic acid) to suppress metabolic inflammation (88), therefore, the local availability of certain lipidic precursors may impact on Treg-mediated control of metabolic inflammation.

The liver is the main organ regulating systemic lipid metabolism and is susceptible to the development of abnormal lipid accumulation and inflammation (steatohepatitis) in pathological conditions induced for instance by high dietary fat intake. Less is known about liver-resident and liver-infiltrating Tregs compared to other tissues. In the early post-natal life of mice, a wave of hepatic Treg colonization occurs that seems dependent on microbiome (91). Of note, the transcriptomic profile of these hepatic neonatal Tregs reveals a high expression of PPARγ and the involvement of lipid handling machinery and oxidative phosphorylation (92). Interestingly, we have observed that such post-natal hepatic Treg expansion was higher in the Mdr2^−/−^ mouse model (spontaneously developing cholangitis and chronic liver disease with time) and was accompanied by an intracellular lipid accumulation in Tregs (93). In adult mice, the identity of liver-resident Tregs remains more elusive with respect to other tissues (94), also because of the significant proportion of Tregs entering this highly vascularized tissue through the blood vessels (95). Like in the adipose tissue, also in the liver Tregs control metabolic inflammation and therefore systemic metabolism. Indeed, post-natal Treg depletion provokes the spontaneous development of steatohepatitis (92); if genetically susceptible adult mice are deprived of Tregs, the hepatic catabolism of lipoproteins is impaired, resulting in hypercholesterolemia and exacerbated atherosclerotic disease (96).

Taken together, the findings in VAT and in liver speak in favor of an inverse relation between external lipid overload and Treg cell-intrinsic lipid accumulation. Indeed, Tregs seem to accumulate lipids, under a PPARγ-driven program and as a consequence of synthesis and/or capture, concomitantly to their own expansion and thus to the control of metabolic inflammation. Conversely, Tregs show impaired lipid metabolism and proliferation in conditions of systemic and local lipid overload. The exact connections between Treg cell-extrinsic and -intrinsic lipid metabolism, and their consequences for Treg suppression and metabolic diseases, have not been clarified. Since Tregs exert several non-immune functions involved in tissue homeostasis, regeneration and repair (97), we cannot exclude that Tregs directly instruct tissue cells for specific metabolic activities, and that the bidirectional crosstalk may be mediated by conventional signaling molecules like amphiregulin (98) or by metabolites, stress signals, and nutrients derived from tissue cells and systemic circulation.

## Treg Expansion Under Metabolic Restriction: the Tumor Microenvironment

A hallmark of the tumor identity is represented by the ability evolved by tumor cells to escape immune recognition, and especially to suppress T cell response. Among the mechanisms concurring to this outcome, the local accumulation of Tregs plays an essential role. It is well-established that Treg frequency increases markedly at the tumor site in most solid malignancies and in both experimental models and cancer patients. Several mechanisms may be involved in this local expansion, including the proliferation of preexisting tissue-resident Tregs, the recruitment of Tregs from the circulation, and the conversion of conventional T cells into pTregs (5). Therefore, the pool of tumor-infiltrating Tregs consists of a mixed population of tTregs and pTregs, possibly recognizing different antigen repertoires, performing specific activities, and showing diverse susceptibility to local signals of proliferation and stabilization (99). Most Tregs at the tumor site display an effector phenotype characterized by high expression of molecules related to their suppressive function and heightened inhibitory activities *ex vivo*. The transcriptomic profile of tumor-infiltrating Tregs results from the combination of tissue-associated signatures with a tumor-specific signature that is shared among different cancer types, and includes costimulatory molecules and chemokine receptors (100). Since the pioneer studies of North and Bursuker on the so-called at that time “suppressor cells” (101), many other studies have clearly demonstrated that Tregs could suppress anti-tumor immunity in experimental models, especially in immunogenic tumors and in certain therapeutic windows. Indeed, CD25+ T cell depletion by means of a specific monoclonal antibody (102), or the inducible genetic ablation of Foxp3+ Tregs (103), could evoke anti-tumor immunity that controlled tumor growth. In the majority of human cancers, a high density of Tregs at the tumor site correlates with a poor prognosis, a finding that confirms the detrimental role of these cells in the battle between host immunity and tumor cells (4). A growing amount of data demonstrate that Tregs play a range of non-immune, tissue-repairing functions that involve the release of amphiregulin, a ligand of epidermal growth factor receptor. Recent work has demonstrated that tissue-infiltrating activated Tregs promote malignancy also through the direct stimulation of epithelial cell growth via amphiregulin (98). Several strategies have been proposed, and have also been tested in some cases, to achieve Treg depletion or functional inactivation in the context of cancer immunotherapy. While CD25-targeted approaches have shown limited success (5), recent studies have highlighted that Tregs are major off-targets of the classical immune checkpoint blockers, being especially sensitive to anti-CTLA-4 antibodies capable of inducing antibody-dependent cell-mediated cytotoxicity (6).

From a metabolic point of view, the tumor microenvironment represents a peculiar and extremely complex context, when multiple metabolic interactions between tumor cells and stromal cells can be established that have not been completely elucidated. The idea that tumor cells evolve the ability to support their proliferative burst by pushing glycolysis was described by Otto Warburg and coworkers in the 1920s and was thus called the Warburg effect. Since then, researchers have accumulated a huge amount of information about the numerous metabolic routes that, together with glycolysis, characterize tumor cell proliferation and survival (104, 105). Besides tumor cells, several stromal and immune cells in the tumor microenvironment are able to reprogram their cellular metabolism when adapting to this peculiar context: for instance, a metabolic crosstalk is established between tumor cells and tumor-associated macrophages, which shapes macrophage functions and finally leads to tumor promotion (106).

This microenvironment poses a series of metabolic hurdles for T cells: (i) hypoxia in poorly vascularized tumor areas can affect T cell functions also through HIF1α induction; (ii) lactate released by both tumor and stromal cells, and the consequent extracellular acidity, can profoundly suppress the effector functions of T cells and compromise anti-tumor immunity; and (iii) the capture of nutrients (glucose, amino acids, and fatty acids) by tumor and stromal cells generates a status of metabolic restriction that ultimately leads to T cell starvation (107). In more detail, competition for glucose between T cells and tumor cells has been identified as a key event in determining the success of anti-tumor T cell activation, through the activity of a glycolytic intermediate, phosphoenolpyruvate, that directly modulates calcium flux downstream TCR signaling (108, 109). Not only glycolysis, but also mitochondrial metabolism is crucial for optimal anti-tumor T cell functions; indeed, a defective mitochondrial biogenesis and oxidative metabolism correlated with a functionally exhausted phenotype (110).

**Figure 5:**
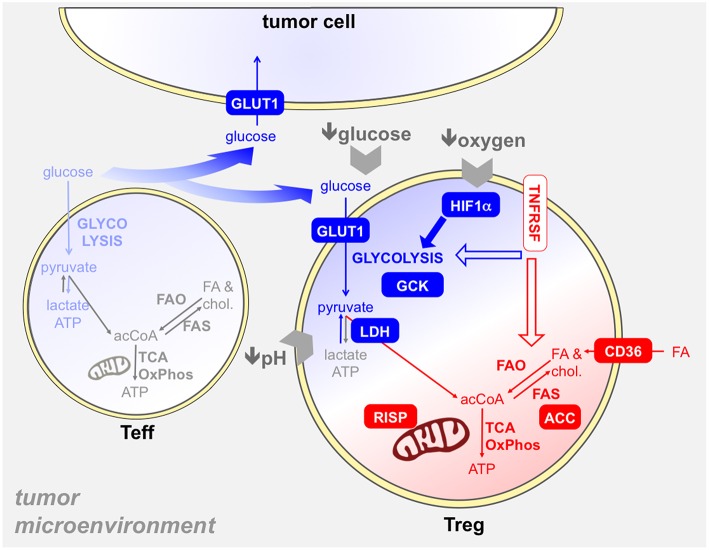
Several metabolic mechanisms may provide an advantage to Tregs in the tumor microenvironment. The tumor microenvironment poses several hurdles to T cells, like hypoxia, glucose restriction, and acidosis. Tregs resist to these obstacles through several mechanisms, which involve both glycolysis (blue) and/or mitochondrial metabolism (red). First, intratumoral Tregs capture glucose at high rates (93, 109), thanks to GLUT1 upregulation, and display a glycolytic program and activity *ex vivo* (93): thus, Tregs may suppress the glycolytic metabolism in effector T cells (light blue) in the tumor microenvironment also through glucose sequestration (74). The usage of GCK instead of hexokinase in glycolysis may endow Tregs to resist in a low-glucose environment (69). Hypoxia triggers HIF1α activation in Tregs, which promotes glycolysis and thus indirectly fosters the oxidation of fatty acids captured through CD36 and other translocators (68). Tregs are more resistant to lactate overload, based on their ability to convert lactate into pyruvate thanks to the modulation of LDH by Foxp3 (40). Mitochondrial metabolism plays a key role in tumor-Treg expansion and function: indeed, mitochondrial complex III (RISP) dictates the ability of Tregs to suppress anti-tumor immunity (66). We have found that intratumoral effector Tregs, expressing TNFR superfamily members, performed fatty acid synthesis through ACC and this pathway was involved in their expansion and function (93). Whether TNFRSF signals can impact on glycolytic and/or mitochondrial metabolism in Tregs remains to be elucidated.

Based on this evidence, the selection of Tregs in the tumor microenvironment may derive from their ability to evolve a metabolic reprogramming that allows their survival and proliferation in such a hostile setting. Several studies have addressed this possibility and have tried to characterize those metabolic pathways that were responsible for tumor-associated Treg expansion and function (Figure 5).

Some data point to a key role of glycolysis in Treg proliferation in the tumor setting. First, intra-tumoral Tregs display particularly high glucose uptake compared to other cell types in a mouse model of melanoma (109), and we have shown that Tregs were able to efficiently compete for glucose with effector T cells in another mouse tumor model (93). Our data also indicate that, compared to effector T cells, tumor-infiltrating Tregs display higher levels of GLUT1 on the cell surface, express higher levels of glycolysis-related genes, and engage a higher glycolytic flux as measured in terms of extracellular acidification rate directly *ex vivo*; in human hepatocellular carcinoma, the gene expression profile of effector Tregs (selected as OX40-positive cells) is significantly enriched in glycolysis-related genes (93). Others have shown that pretreatment of human Tregs with a TLR8 agonist (that inhibits glycolysis), or 2-DG, reversed their ability to control anti-tumor T cell response *in vivo*, in a humanized mouse model (73). Of note, the pretreatment impaired Treg ability to induce senescence in responder CD8 T cells. Indeed, competition for glucose is a major trigger for T cell senescence, and Tregs may cooperate with tumor cells in consuming glucose and starving effector T cells in the tumor microenvironment (74). It has been proposed that, in low-glucose contexts such as the tumor site, Tregs may utilize glucokinase instead of hexokinase to perform glycolysis, having the former a much lower affinity for glucose (69). Altogether, these results suggest that Tregs possess the machineries needed for capturing and utilizing glucose in the tumor context, and that glycolysis may be involved in the maintenance of the tumor-associated Treg population *in vivo*.

More elusive is the role of oxidative phosphorylation in proliferation and survival of tumor-infiltrating Tregs. We have observed that Tregs freshly extracted from murine tumors consume oxygen at similar rates as effector T cells (93). Others have shown that mice, where mitochondrial complex III is ablated specifically in Tregs, are protected from tumor growth; however, this effect may be ascribed also to the epigenetic effect of mitochondrial metabolites, besides the defect in mitochondrial respiration (66).

Foxp3 has been shown to directly shift the glycolytic into oxidative metabolism: this program also includes the peculiar ability of Tregs to oxidize lactate into pyruvate also in normal conditions, whereas Tconvs show this activity only in low-glucose, high-lactate conditions. However, Tconvs and Tregs are differentially susceptible to lactate overload and the consequent oxidation through the enzyme LDH: Tconvs rely primarily on glycolysis for their activation, and NAD depletion during lactate oxidation prevents GAPDH activity and suppresses their proliferation; conversely, Tregs are more resistant to the suppressive effect of lactate, possibly being less glycolysis-dependent for their activation, and containing higher levels of NAD continuously regenerated during oxidative phosphorylation (40). These data suggest that in low-glucose, high-lactate conditions, like in the tumor microenvironment, Tregs may have a selective metabolic advantage.

Fatty acids can become a major substrate for oxidative phosphorylation in some conditions. It has been shown that, in hypoxic settings, HIF1α activation diverts glucose away from mitochondria, leaving fatty acids as the main oxidative substrate for Tregs. Therefore, in hypoxic areas of the tumor microenvironment, Tregs may capture and utilize lipids to perform their metabolic functions: accordingly, intra-tumoral Tregs were found to express high levels of the fatty acid transporters CD36, SLC27A1, and SLC27A4, and to perform fatty acid uptake *in vivo*, in a mouse model of glioma (68). In this model, fatty acid oxidation seemed required for Treg suppression: indeed, etomoxir administration to tumor-bearing mice reduced tumor growth while reducing Treg frequencies (68). It has been proposed that the ability of Tregs to oxidize fatty acids may protect them from lipotoxicity (44), thus endowing Tregs with a further metabolic advantage over effector T cells.

In a different mouse tumor model, we have observed that intra-tumoral Tregs accumulated neutral fatty acids, an event that was not related to fatty acid capture but rather derived from fatty acid synthesis (93). The expression of genes related to this pathway, the metabolite profile, the sensitivity of Treg to specific inhibitors of fatty acid synthesis, and the observation that 2-DG prevents fatty acid accumulation *in vitro* corroborate the hypothesis that tumor-infiltrating Tregs utilize glucose not only as a supply of energy but also as a source of precursors to build macromolecules like fatty acids. We have started to appreciate that many macromolecules can exert several non-energetic and non-structural activities. For instance, fatty acids represent a preferential source of acetyl groups for histone acetylation (111), and thus fatty acid supply or biosynthesis may impact the epigenetic profile of the cells.

## Concluding Remarks

Even though some controversies still remain regarding the metabolic requirements of specific events during the life of a Treg, some general conclusions can be drawn from the existing data. First of all, the view that Tregs are oxidative cells need to be carefully revised; indeed, this assumption mostly comes from studies addressing the development of Tregs *in vitro*, rather than the *in vivo* dynamics of established Tregs. Moreover, many studies are also affected by experimental limitations such as the usage of TGFβ-based protocols and of pharmacological inhibitors with many off-target effects. Based on pieces of evidence coming from many studies, it is clear that Tregs can use different metabolic pathways, including glycolysis, in different phases of their life and in different activities. Generally speaking, while mTOR-driven glycolysis-lipogenesis seems required for Treg development and migration, Foxp3-driven lipolytic-oxidative metabolism is more strictly related to Treg suppressive function. Whether the two axes must coexist in the same Treg cell to achieve a full immune regulatory activity or can be segregated into distinct Treg subtypes or distinct phases of Treg activities, remains to be clarified.

The capacity of Tregs to switch through different metabolic programs may render them particularly able to adapt to hostile microenvironments, and the tumor may represent a prototypical context where Treg adaptation occurs: here, Tregs may be positively selected based on their ability to compete efficiently for glucose, to use alternative glycolytic enzymes, to capture and catabolize lipids thus avoiding incidental lipotoxicity, to better resist to high lactate exposure, and many other mechanisms still to be discovered. Among the signals in the tumor microenvironment supporting metabolic activation in Tregs, the TNFR-related signals may play key roles that have been only incompletely appreciated; these receptors are highly expressed by Tregs at the tumor site from many different tumor histotypes and may drive a switch toward an effector phenotype that includes immunological as well as metabolic features.

Targeting metabolism is considered a promising therapeutic approach for cancer therapy. However, it is now well-established that appropriately designed metabolic interventions can profoundly reshape immune cell functions and rescue anti-tumor immunity (112). Therefore, a holistic assessment of the metabolism of stromal and immune cells, together with tumor cells, would allow a more accurate design of future therapeutic strategies for cancer treatment (112). It should be considered that “metabolic drugs” targeting specific cell types in tumor-bearing hosts are not currently available, thus we are still far from a cell-directed metabolic intervention that would selectively inhibit detrimental immune cells and tumor cells while sparing normal cells. However, it could be predicted that some of these drugs could, at least preferentially, target metabolically active cells at the tumor site. According to this view, Tregs may be preferentially susceptible to metabolic interventions in tumors because of their relative abundance at the tumor site and of their stronger metabolic activation compared to other infiltrating T cells.

## Author Contributions

IP and SP conceived the concepts behind this review, wrote the manuscript, and prepared the figures.

### Conflict of Interest Statement

The authors declare that the research was conducted in the absence of any commercial or financial relationships that could be construed as a potential conflict of interest.
